# 3,4-Dimeth­oxy­benzaldehyde [2,8-bis­(trifluoro­meth­yl)quinolin-4-yl]hydrazone

**DOI:** 10.1107/S1600536810021616

**Published:** 2010-06-23

**Authors:** Waleed Fedl Ali Al-eryani, J. Shylaja Kumari, H. K. Arunkashi, Suresh Babu Vepuri, H. C. Devarajegowda

**Affiliations:** a Department of Physics, Yuvaraja’s College (Constituent College), University of Mysore, Mysore 570 005, Karnataka, India; b Department of Physics, AVK College for Women, Hassan 573 201, Karnataka, India; c Department of Pharmaceutical Chemistry, GITAM Institute of Pharmacy, GITAM University, Visakhapatnam 530 045, Andhrapradesh, India

## Abstract

In the title compound, C_20_H_15_F_6_N_3_O_2_, the quinoline ring system is almost coplanar with the benzene ring; the dihedral angle between the two planes is 2.31 (8)°. The crystal structure displays an inter­molecular C—H⋯F hydrogen bond. In addition, a weak π–π inter­action is observed between the unfused benzene ring and the benzene ring of quinoline, with a centroid–centroid distance of 3.586 (1) Å.

## Related literature

For general background to quinolines, see: Mao *et al.* (2009 ▶); Bermudez *et al.*(2004 ▶); Jayaprakash *et al.* (2006 ▶); Andries *et al.* (2005 ▶). For related structures, see: Skörska *et al.* (2005 ▶).
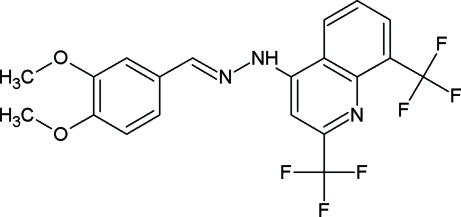


## Experimental

### 

#### Crystal data

C_20_H_15_F_6_N_3_O_2_
*M* *_r_* = 443.35
Triclinic, 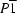

*a* = 7.0359 (6) Å
*b* = 8.9617 (8) Å
*c* = 15.5315 (14) Å
α = 90.154 (1)°
β = 93.951 (1)°
γ = 96.449 (1)°
*V* = 970.75 (15) Å^3^
*Z* = 2
Mo *K*α radiation
μ = 0.14 mm^−1^
*T* = 298 K
0.22 × 0.15 × 0.12 mm

#### Data collection

Bruker SMART CCD area-detector diffractometer
Absorption correction: ψ scan (*SADABS*; Sheldrick, 2007 ▶) *T*_min_ = 0.975, *T*_max_ = 0.984
9677 measured reflections
3756 independent reflections
2951 reflections with *I* > 2σ(*I*)
*R*_int_ = 0.023

#### Refinement

*R*[*F* ^2^ > 2σ(*F* ^2^)] = 0.060
*wR*(*F* ^2^) = 0.175
*S* = 1.03
3756 reflections
281 parameters
H-atom parameters constrained
Δρ_max_ = 0.51 e Å^−3^
Δρ_min_ = −0.31 e Å^−3^

### 

Data collection: *SMART* (Bruker, 2001 ▶); cell refinement: *SAINT* (Bruker, 2001 ▶); data reduction: *SAINT*; program(s) used to solve structure: *SHELXS97* (Sheldrick, 2008 ▶); program(s) used to refine structure: *SHELXL97* (Sheldrick, 2008 ▶); molecular graphics: *ORTEP-3* (Farrugia, 1997 ▶); software used to prepare material for publication: *SHELXL97*.

## Supplementary Material

Crystal structure: contains datablocks I, global. DOI: 10.1107/S1600536810021616/wn2391sup1.cif

Structure factors: contains datablocks I. DOI: 10.1107/S1600536810021616/wn2391Isup2.hkl

Additional supplementary materials:  crystallographic information; 3D view; checkCIF report

## Figures and Tables

**Table 1:** Hydrogen-bond geometry (Å, °)

*D*—H⋯*A*	*D*—H	H⋯*A*	*D*⋯*A*	*D*—H⋯*A*
C12—H12⋯F1^i^	0.93	2.47	3.387 (3)	168

Symmetry code: (i) 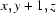.

**Fig. 1.**
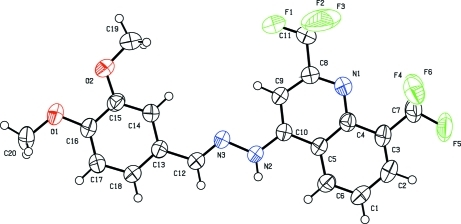
The molecular structure of the title compound. Displacement ellipsoids are drawn at the 50% probability level. Hydrogen atoms are shown as spheres of arbitrary radius.

**Fig. 2.**
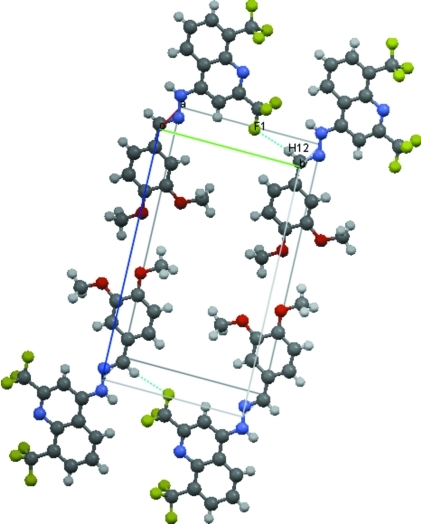
Packing of the molecules showing hydrogen bonds as dashed lines.

## supplementary crystallographic information

### Comment

2,8-Bis(trifluoromethyl)quinolin-4-yl]-(2-piperidyl)methanol (mefloquin) is a popular antimalarial drug. Further, studies have reported that it also possesses important structural features required for antimicrobial activity (Mao *et al.* 2009; Bermudez *et al.* 2004; Jayaprakash *et al.* 2006). Quinoline is the essential structural feature found in mefloquin and recently developed antimycobacterial drugs (Andries *et al.* 2005). Thus, quinoline derivatives are good lead molecules to further develop drug candidates against mycobacterium tuberculosis and as antibacterial agents. On the basis of these observations we have synthesized a quinoline derivative, in which a hydrazone group has been attached at the 4 position of the mefloquin structure, expecting that the newly designed molecule would exhibit some antibacterial activity. In this paper we report the crystal structure of 3,4-dimethoxybenzaldehyde [2,8-bis(trifluoromethyl)quinolin-4-yl]hydrazone.

The asymmetric unit of the title compound contains one molecule (Fig. 1). A very small dihedral angle [2.31 (8)°] between the quinoline system and the benzene ring indicates that these two systems are coplanar. In the crystal structure, intermolecular C12—H12···F1 hydrogen bonding (Table 1) involving the trifluoromethyl and methine groups results in the formation of a three-dimensional ladder-type network (Fig.2). In addition, a weak π-π interaction is observed between the benzene ring (C1-C6) and benzene ring (C13-C18) of quinoline, with a centroid-centroid distance of 3.586 (1) Å.

The crystal structures of the mefloquine base and its salt complexes have been reported (Skörska *et al.* 2005). However, these are only related to the quinoline portion of our structure.

### Experimental

A mixture of [2,8-bis(trifluoromethyl)quinolin-4-yl]hydrazine (10 mmol) and 3,4 trimethoxy benzaldehyde (10 mmol) in glacial acetic acid (50 ml) was heated at reflux for 3 h. The reaction mixture was concentrated under reduced pressure, cooled, and the resulting solid hydrazone was filtered, washed with water and cold ethanol. The crude product was purified by column chromatography. Crystals suitable for X-ray analysis were obtained by dissolving the pure compound in hot methanol and slow evaporation of the solvent at room temperature. Yield:  72%, Mp. 487 K.

### Refinement

All H atoms were placed at calculated positions; N—H = 0.86 Å, C—H = 0.93 Å for aromatic H, 0.96 Å for methyl H and refined using a riding model with *U*_iso_(H) = 1.5*U*_eq_(C) for methyl H and *U*_iso_(H) = 1.2*U*_eq_(C,N) for the imine H and all other carbon-bound H atoms.

### Crystal data

**Table:**

C_20_H_15_F_6_N_3_O_2_	*Z* = 2
*M**_r_* = 443.35	*F*(000) = 452
Triclinic, *P*1	*D*_x_ = 1.517 Mg m^−^^3^
Hall symbol: -P 1	Melting point: 487 K
*a* = 7.0359 (6) Å	Mo *K*α radiation, λ = 0.71073 Å
*b* = 8.9617 (8) Å	Cell parameters from 3756 reflections
*c* = 15.5315 (14) Å	θ = 2.3–25.9°
α = 90.154 (1)°	µ = 0.14 mm^−^^1^
β = 93.951 (1)°	*T* = 298 K
γ = 96.449 (1)°	Plate, colourless
*V* = 970.75 (15) Å^3^	0.22 × 0.15 × 0.12 mm

### Data collection

**Table:**

Bruker SMART CCD area-detector diffractometer	3756 independent reflections
Radiation source: fine-focus sealed tube	2951 reflections with *I* > 2σ(*I*)
graphite	*R*_int_ = 0.023
ω and φ scans	θ_max_ = 25.9°, θ_min_ = 2.3°
Absorption correction: ψ scan (*SADABS*; Sheldrick, 2007)	*h* = −8→8
*T*_min_ = 0.975, *T*_max_ = 0.984	*k* = −11→11
9677 measured reflections	*l* = −19→19

### Refinement

**Table:**

Refinement on *F*^2^	Secondary atom site location: difference Fourier map
Least-squares matrix: full	Hydrogen site location: inferred from neighbouring sites
*R*[*F*^2^ > 2σ(*F*^2^)] = 0.060	H-atom parameters constrained
*wR*(*F*^2^) = 0.175	*w* = 1/[σ^2^(*F*_o_^2^) + (0.0861*P*)^2^ + 0.4197*P*] where *P* = (*F*_o_^2^ + 2*F*_c_^2^)/3
*S* = 1.03	(Δ/σ)_max_ < 0.001
3756 reflections	Δρ_max_ = 0.51 e Å^−^^3^
281 parameters	Δρ_min_ = −0.31 e Å^−^^3^
0 restraints	Extinction correction: *SHELXL97* (Sheldrick, 2008), Fc^*^=kFc[1+0.001xFc^2^λ^3^/sin(2θ)]^-1/4^
Primary atom site location: structure-invariant direct methods	Extinction coefficient: 0.013 (3)

### Special details

**Table:**

Experimental. 1H NMR (300 MHz, CD3 OD): δ, 3.886 (s, 3H, OCH3), 3.930 (s, 3H, OCH3) 7.047-7.019 (d, IH, 3,4-dimethoxyphenyl, J = 8.1Hz) 7.297-7.269 (t, 1H, trifluoromethylquinoline, J = 8.4Hz), 7.46 (s, IH, trifluoromethylquinoline), 7.667-7.640 (d, 1H, 3,4-dimethoxyphenyl, J=8.1Hz), 8.138-8.114 (d, 1H, trifluoromethylquinoline, J=7.2Hz), 8.26 (s, 1H, =CH), 8.263 (s, IH, 3,4-dimethoxyphenyl), 8.527-8.498 (d, IH, trifluoromethylquinoline, J=8.7Hz), 8.52 (s, 1H, N-H).
Geometry. All e.s.d.'s (except the e.s.d. in the dihedral angle between two l.s. planes) are estimated using the full covariance matrix. The cell e.s.d.'s are taken into account individually in the estimation of e.s.d.'s in distances, angles and torsion angles; correlations between e.s.d.'s in cell parameters are only used when they are defined by crystal symmetry. An approximate (isotropic) treatment of cell e.s.d.'s is used for estimating e.s.d.'s involving l.s. planes.
Refinement. Refinement of *F*^2^ against ALL reflections. The weighted *R*-factor *wR* and goodness of fit *S* are based on *F*^2^, conventional *R*-factors *R* are based on *F*, with *F* set to zero for negative *F*^2^. The threshold expression of *F*^2^ > σ(*F*^2^) is used only for calculating *R*-factors(gt) *etc*. and is not relevant to the choice of reflections for refinement. *R*-factors based on *F*^2^ are statistically about twice as large as those based on *F*, and *R*- factors based on ALL data will be even larger.

### Fractional atomic coordinates and isotropic or equivalent isotropic displacement parameters (Å^2^)

**Table:**

	*x*	*y*	*z*	*U*_iso_*/*U*_eq_	
F1	0.2412 (5)	0.4438 (2)	1.01640 (13)	0.1566 (13)	
F2	0.1191 (7)	0.3559 (3)	1.1239 (2)	0.211 (2)	
F3	0.4056 (6)	0.3784 (3)	1.1201 (2)	0.1887 (16)	
F4	0.1562 (3)	0.5209 (2)	1.39246 (11)	0.0901 (5)	
F5	0.3417 (3)	0.6419 (2)	1.48883 (9)	0.0978 (6)	
F6	0.4605 (3)	0.5329 (2)	1.38655 (10)	0.0913 (6)	
O1	0.1621 (3)	1.07781 (19)	0.57898 (9)	0.0638 (5)	
O2	0.1874 (3)	0.83177 (18)	0.65840 (10)	0.0688 (5)	
N1	0.2843 (3)	0.6043 (2)	1.22381 (12)	0.0578 (5)	
N2	0.2663 (3)	0.98738 (19)	1.07163 (10)	0.0507 (5)	
H2	0.2733	1.0750	1.0951	0.061*	
N3	0.2477 (3)	0.97088 (19)	0.98344 (10)	0.0482 (4)	
C1	0.3304 (3)	1.0213 (3)	1.34583 (14)	0.0580 (6)	
H1	0.3429	1.1138	1.3742	0.070*	
C2	0.3326 (3)	0.8892 (3)	1.39354 (14)	0.0582 (6)	
H2A	0.3449	0.8943	1.4535	0.070*	
C3	0.3169 (3)	0.7533 (3)	1.35271 (13)	0.0525 (5)	
C4	0.2979 (3)	0.7427 (2)	1.26103 (13)	0.0470 (5)	
C5	0.2936 (3)	0.8770 (2)	1.21338 (12)	0.0436 (5)	
C6	0.3100 (3)	1.0148 (2)	1.25823 (13)	0.0518 (5)	
H6	0.3069	1.1034	1.2273	0.062*	
C7	0.3190 (4)	0.6124 (3)	1.40417 (15)	0.0689 (7)	
C8	0.2689 (4)	0.6011 (2)	1.13901 (14)	0.0591 (6)	
C9	0.2625 (3)	0.7229 (2)	1.08485 (13)	0.0538 (5)	
H9	0.2509	0.7099	1.0252	0.065*	
C10	0.2736 (3)	0.8634 (2)	1.12140 (12)	0.0444 (5)	
C11	0.2571 (6)	0.4467 (3)	1.09982 (19)	0.0922 (11)	
C12	0.2377 (3)	1.0891 (2)	0.94007 (13)	0.0506 (5)	
H12	0.2427	1.1804	0.9692	0.061*	
C13	0.2186 (3)	1.0878 (2)	0.84631 (12)	0.0450 (5)	
C14	0.2105 (3)	0.9538 (2)	0.79899 (13)	0.0478 (5)	
H14	0.2167	0.8636	0.8279	0.057*	
C15	0.1934 (3)	0.9543 (2)	0.71040 (13)	0.0492 (5)	
C16	0.1818 (3)	1.0908 (2)	0.66624 (12)	0.0478 (5)	
C17	0.1917 (3)	1.2227 (2)	0.71299 (13)	0.0521 (5)	
H17	0.1863	1.3134	0.6845	0.063*	
C18	0.2096 (3)	1.2204 (2)	0.80233 (13)	0.0514 (5)	
H18	0.2156	1.3101	0.8332	0.062*	
C19	0.2110 (5)	0.6924 (3)	0.69794 (18)	0.0804 (8)	
H19A	0.2042	0.6154	0.6544	0.121*	
H19B	0.1113	0.6681	0.7365	0.121*	
H19C	0.3335	0.6989	0.7298	0.121*	
C20	0.1384 (4)	1.2097 (3)	0.53018 (14)	0.0654 (7)	
H20A	0.1262	1.1847	0.4698	0.098*	
H20B	0.2480	1.2825	0.5421	0.098*	
H20C	0.0251	1.2506	0.5458	0.098*	

### Atomic displacement parameters (Å^2^)

**Table:**

	*U*^11^	*U*^22^	*U*^33^	*U*^12^	*U*^13^	*U*^23^
F1	0.342 (4)	0.0590 (11)	0.0692 (12)	0.0322 (16)	0.0014 (16)	−0.0190 (9)
F2	0.345 (5)	0.0832 (16)	0.196 (3)	−0.085 (2)	0.128 (3)	−0.0574 (17)
F3	0.282 (4)	0.1017 (18)	0.190 (3)	0.103 (2)	−0.062 (3)	−0.0563 (18)
F4	0.0978 (12)	0.0869 (12)	0.0843 (11)	0.0028 (9)	0.0072 (9)	0.0326 (9)
F5	0.1344 (16)	0.1228 (15)	0.0411 (8)	0.0407 (12)	−0.0016 (8)	0.0207 (8)
F6	0.1058 (13)	0.1033 (13)	0.0741 (10)	0.0536 (10)	0.0027 (9)	0.0238 (9)
O1	0.0832 (11)	0.0717 (11)	0.0365 (8)	0.0125 (8)	−0.0023 (7)	0.0003 (7)
O2	0.1029 (13)	0.0560 (9)	0.0466 (9)	0.0126 (9)	−0.0081 (8)	−0.0122 (7)
N1	0.0775 (13)	0.0495 (10)	0.0471 (10)	0.0120 (9)	0.0014 (9)	0.0041 (8)
N2	0.0750 (12)	0.0433 (9)	0.0338 (9)	0.0082 (8)	0.0023 (8)	−0.0030 (7)
N3	0.0617 (11)	0.0477 (9)	0.0352 (9)	0.0071 (8)	0.0016 (7)	−0.0013 (7)
C1	0.0635 (14)	0.0646 (14)	0.0457 (12)	0.0051 (11)	0.0053 (10)	−0.0152 (10)
C2	0.0559 (13)	0.0826 (16)	0.0356 (10)	0.0082 (11)	−0.0001 (9)	−0.0064 (10)
C3	0.0480 (11)	0.0712 (14)	0.0388 (11)	0.0114 (10)	−0.0001 (8)	0.0043 (10)
C4	0.0475 (11)	0.0536 (12)	0.0403 (10)	0.0092 (9)	−0.0003 (8)	0.0019 (8)
C5	0.0433 (10)	0.0494 (11)	0.0381 (10)	0.0061 (8)	0.0015 (8)	−0.0027 (8)
C6	0.0580 (12)	0.0523 (12)	0.0450 (11)	0.0053 (9)	0.0053 (9)	−0.0043 (9)
C7	0.0754 (17)	0.0861 (18)	0.0475 (13)	0.0230 (15)	−0.0017 (11)	0.0141 (12)
C8	0.0836 (16)	0.0457 (12)	0.0479 (12)	0.0085 (11)	0.0013 (11)	−0.0017 (9)
C9	0.0763 (15)	0.0484 (12)	0.0363 (10)	0.0077 (10)	−0.0004 (9)	−0.0035 (8)
C10	0.0492 (11)	0.0457 (11)	0.0382 (10)	0.0059 (8)	0.0003 (8)	0.0009 (8)
C11	0.172 (4)	0.0439 (14)	0.0622 (17)	0.0193 (18)	0.0067 (18)	0.0032 (12)
C12	0.0666 (13)	0.0453 (11)	0.0404 (11)	0.0091 (9)	0.0034 (9)	−0.0034 (9)
C13	0.0497 (11)	0.0479 (11)	0.0376 (10)	0.0065 (8)	0.0027 (8)	−0.0005 (8)
C14	0.0542 (12)	0.0468 (11)	0.0419 (11)	0.0063 (9)	−0.0002 (9)	0.0019 (8)
C15	0.0518 (11)	0.0524 (12)	0.0426 (11)	0.0054 (9)	−0.0032 (9)	−0.0067 (9)
C16	0.0485 (11)	0.0588 (12)	0.0361 (10)	0.0078 (9)	0.0006 (8)	−0.0006 (9)
C17	0.0624 (13)	0.0510 (12)	0.0438 (11)	0.0101 (10)	0.0037 (9)	0.0079 (9)
C18	0.0664 (13)	0.0465 (11)	0.0422 (11)	0.0096 (9)	0.0049 (9)	−0.0031 (9)
C19	0.125 (3)	0.0507 (14)	0.0645 (16)	0.0107 (14)	0.0008 (15)	−0.0115 (11)
C20	0.0732 (15)	0.0844 (17)	0.0398 (11)	0.0141 (13)	0.0027 (10)	0.0113 (11)

### Geometric parameters (Å, °)

**Table:**

F1—C11	1.293 (3)	C5—C6	1.406 (3)
F2—C11	1.272 (5)	C5—C10	1.429 (3)
F3—C11	1.291 (5)	C6—H6	0.9300
F4—C7	1.333 (3)	C8—C9	1.383 (3)
F5—C7	1.336 (3)	C8—C11	1.502 (3)
F6—C7	1.330 (3)	C9—C10	1.372 (3)
O1—C16	1.356 (2)	C9—H9	0.9300
O1—C20	1.425 (3)	C12—C13	1.453 (3)
O2—C15	1.357 (3)	C12—H12	0.9300
O2—C19	1.415 (3)	C13—C18	1.378 (3)
N1—C8	1.314 (3)	C13—C14	1.399 (3)
N1—C4	1.358 (3)	C14—C15	1.373 (3)
N2—C10	1.359 (3)	C14—H14	0.9300
N2—N3	1.373 (2)	C15—C16	1.412 (3)
N2—H2	0.8600	C16—C17	1.378 (3)
N3—C12	1.263 (3)	C17—C18	1.385 (3)
C1—C6	1.359 (3)	C17—H17	0.9300
C1—C2	1.399 (3)	C18—H18	0.9300
C1—H1	0.9300	C19—H19A	0.9600
C2—C3	1.362 (3)	C19—H19B	0.9600
C2—H2A	0.9300	C19—H19C	0.9600
C3—C4	1.423 (3)	C20—H20A	0.9600
C3—C7	1.497 (3)	C20—H20B	0.9600
C4—C5	1.417 (3)	C20—H20C	0.9600
			
C16—O1—C20	118.09 (18)	F2—C11—F3	103.2 (3)
C15—O2—C19	117.76 (18)	F2—C11—F1	106.1 (3)
C8—N1—C4	115.71 (18)	F3—C11—F1	104.6 (3)
C10—N2—N3	119.15 (16)	F2—C11—C8	114.2 (3)
C10—N2—H2	120.4	F3—C11—C8	113.4 (3)
N3—N2—H2	120.4	F1—C11—C8	114.3 (2)
C12—N3—N2	116.75 (17)	N3—C12—C13	122.43 (19)
C6—C1—C2	120.2 (2)	N3—C12—H12	118.8
C6—C1—H1	119.9	C13—C12—H12	118.8
C2—C1—H1	119.9	C18—C13—C14	118.76 (18)
C3—C2—C1	120.41 (19)	C18—C13—C12	119.92 (18)
C3—C2—H2A	119.8	C14—C13—C12	121.32 (18)
C1—C2—H2A	119.8	C15—C14—C13	120.70 (19)
C2—C3—C4	120.8 (2)	C15—C14—H14	119.7
C2—C3—C7	120.1 (2)	C13—C14—H14	119.7
C4—C3—C7	119.0 (2)	O2—C15—C14	125.5 (2)
N1—C4—C5	123.44 (18)	O2—C15—C16	114.57 (18)
N1—C4—C3	118.27 (19)	C14—C15—C16	119.92 (19)
C5—C4—C3	118.29 (19)	O1—C16—C17	125.84 (19)
C6—C5—C4	118.94 (18)	O1—C16—C15	114.91 (18)
C6—C5—C10	123.79 (19)	C17—C16—C15	119.25 (18)
C4—C5—C10	117.27 (18)	C16—C17—C18	120.15 (19)
C1—C6—C5	121.3 (2)	C16—C17—H17	119.9
C1—C6—H6	119.3	C18—C17—H17	119.9
C5—C6—H6	119.3	C13—C18—C17	121.22 (19)
F6—C7—F4	107.0 (2)	C13—C18—H18	119.4
F6—C7—F5	106.04 (19)	C17—C18—H18	119.4
F4—C7—F5	105.7 (2)	O2—C19—H19A	109.5
F6—C7—C3	113.2 (2)	O2—C19—H19B	109.5
F4—C7—C3	112.9 (2)	H19A—C19—H19B	109.5
F5—C7—C3	111.5 (2)	O2—C19—H19C	109.5
N1—C8—C9	126.8 (2)	H19A—C19—H19C	109.5
N1—C8—C11	114.4 (2)	H19B—C19—H19C	109.5
C9—C8—C11	118.8 (2)	O1—C20—H20A	109.5
C10—C9—C8	118.24 (19)	O1—C20—H20B	109.5
C10—C9—H9	120.9	H20A—C20—H20B	109.5
C8—C9—H9	120.9	O1—C20—H20C	109.5
N2—C10—C9	121.02 (18)	H20A—C20—H20C	109.5
N2—C10—C5	120.47 (18)	H20B—C20—H20C	109.5
C9—C10—C5	118.52 (18)		
			
C10—N2—N3—C12	178.62 (19)	C6—C5—C10—N2	−1.1 (3)
C6—C1—C2—C3	0.9 (3)	C4—C5—C10—N2	179.28 (18)
C1—C2—C3—C4	0.0 (3)	C6—C5—C10—C9	178.8 (2)
C1—C2—C3—C7	−179.9 (2)	C4—C5—C10—C9	−0.8 (3)
C8—N1—C4—C5	0.8 (3)	N1—C8—C11—F2	57.4 (5)
C8—N1—C4—C3	−179.4 (2)	C9—C8—C11—F2	−122.5 (4)
C2—C3—C4—N1	179.5 (2)	N1—C8—C11—F3	−60.4 (4)
C7—C3—C4—N1	−0.7 (3)	C9—C8—C11—F3	119.7 (3)
C2—C3—C4—C5	−0.7 (3)	N1—C8—C11—F1	179.8 (3)
C7—C3—C4—C5	179.15 (19)	C9—C8—C11—F1	−0.1 (5)
N1—C4—C5—C6	−179.6 (2)	N2—N3—C12—C13	179.74 (19)
C3—C4—C5—C6	0.6 (3)	N3—C12—C13—C18	−179.6 (2)
N1—C4—C5—C10	0.1 (3)	N3—C12—C13—C14	−0.1 (3)
C3—C4—C5—C10	−179.77 (18)	C18—C13—C14—C15	−0.2 (3)
C2—C1—C6—C5	−0.9 (3)	C12—C13—C14—C15	−179.7 (2)
C4—C5—C6—C1	0.2 (3)	C19—O2—C15—C14	−3.6 (4)
C10—C5—C6—C1	−179.4 (2)	C19—O2—C15—C16	175.9 (2)
C2—C3—C7—F6	−120.9 (2)	C13—C14—C15—O2	178.9 (2)
C4—C3—C7—F6	59.2 (3)	C13—C14—C15—C16	−0.6 (3)
C2—C3—C7—F4	117.4 (3)	C20—O1—C16—C17	−3.8 (3)
C4—C3—C7—F4	−62.4 (3)	C20—O1—C16—C15	176.45 (19)
C2—C3—C7—F5	−1.5 (3)	O2—C15—C16—O1	1.5 (3)
C4—C3—C7—F5	178.7 (2)	C14—C15—C16—O1	−178.97 (19)
C4—N1—C8—C9	−1.0 (4)	O2—C15—C16—C17	−178.3 (2)
C4—N1—C8—C11	179.1 (3)	C14—C15—C16—C17	1.2 (3)
N1—C8—C9—C10	0.2 (4)	O1—C16—C17—C18	179.2 (2)
C11—C8—C9—C10	−179.9 (3)	C15—C16—C17—C18	−1.0 (3)
N3—N2—C10—C9	−0.1 (3)	C14—C13—C18—C17	0.4 (3)
N3—N2—C10—C5	179.80 (17)	C12—C13—C18—C17	179.9 (2)
C8—C9—C10—N2	−179.4 (2)	C16—C17—C18—C13	0.2 (3)
C8—C9—C10—C5	0.8 (3)		

### Hydrogen-bond geometry (Å, °)

**Table:**

*D*—H···*A*	*D*—H	H···*A*	*D*···*A*	*D*—H···*A*
C12—H12···F1^i^	0.93	2.47	3.387 (3)	168

Symmetry codes: (i) *x*, *y*+1, *z*.
